# Medical consultants’ experience of collective leadership in complexity: a qualitative interview study

**DOI:** 10.1108/JHOM-04-2023-0104

**Published:** 2024-08-19

**Authors:** Áine Carroll, Jane McKenzie, Claire Collins

**Affiliations:** School of Medicine, University College Dublin, Dublin, Ireland; National Rehabilitation Hospital, Dublin, Ireland; Henley Business School, University of Reading, Reading, UK

**Keywords:** Leadership, Complexity, Health and social care, Inductive thematic analysis

## Abstract

**Purpose:**

The aim of this study was to explore and understand the leadership experiences of medical consultants prior to a major hospital move. Health and care is becoming increasingly complex and there is no greater challenge than the move to a new hospital. Effective leadership has been identified as being essential for successful transition. However, there is very little evidence of how medical consultants experience effective leadership.

**Design/methodology/approach:**

A qualitative methodology was utilized with one-to-one semi-structured interviews conducted with ten medical consultants. These were transcribed verbatim and analyzed using inductive thematic analysis. The research complied with the consolidated criteria for reporting qualitative research (COREQ).

**Findings:**

Four themes were found to influence medical consultants’ experience of leadership: collaboration, patient centredness, governance and knowledge mobilization. Various factors were identified that negatively influenced their leadership effectiveness. The findings suggest that there are a number of factors that influence complexity leadership effectiveness. Addressing these areas may enhance leadership effectiveness and the experience of leadership in medical consultants.

**Research limitations/implications:**

This study provides a rich exploration of medical consultants’ experience of collective leadership prior to a transition to a new hospital and provides new understandings of the way collective leadership is experienced in the lead up to a major transition and makes recommendations for future leadership research and practice.

**Practical implications:**

The findings suggest that there are a number of factors that influence complexity leadership effectiveness. Addressing these areas may enhance leadership effectiveness and the experience of leadership in medical consultants.

**Social implications:**

Clinical leadership is associated with better outcomes for patients therefore any interventions that enhance leadership capability will improve outcomes for patients and therefore benefit society.

**Originality/value:**

This is the first research to explore medical consultants’ experience of collective leadership prior to a transition to a new hospital.

## Introduction

It is increasingly being recognized that health and care systems are complex systems (Braithwaite *et al.*, 2017; Carroll *et al.*, 2021; Rusoja *et al*., 2018; Thompson *et al.*, 2016). Complex systems have many interacting elements that exhibit self-organization, systemic phenomena, path dependency, sensitivity to context, emergence and episodicity (Boulton *et al.*, 2015). In complex systems, individuals and teams need to work together and learn together to produce innovation and adaptation (Cullen-Lester and Yammarino, 2016; Lichtenstein *et al.*, 2006). The move to a new hospital facility is an unusual, complex, and significant event. There are few organizational events that can compare to the change process associated with the transition to a new hospital facility (Collado, 2021). As well as the intricacies of the physical build, such moves have been shown to generate significant challenges for staff (Slosberg *et al.*, 2018). Research has shown that a move to a new facility can negatively impact on staff satisfaction and retention and therefore leadership and teamwork, planning and learning are required to ensure a successful transition (Berry and Parish, 2008). Studies have demonstrated that organizations that have high levels of medical engagement have better organizational performance, patient satisfaction and morbidity (Dickinson *et al.*, 2013; Hamilton *et al.*, 2008; Clay-Williams *et al.*, 2017). However, research has also shown that in many healthcare systems doctors are dissatisfied and, in some cases, alienated from the systems and organizations in which they work (Smith, 2001).

### Literature review and theoretical perspective

Leadership has been identified as a key catalyst for successful change and positive culture (Yukl, 2012, West *et al.*, 2014a, b) and research has clearly demonstrated that physician leaders are associated with better organizational performance and outcomes (Goodall, 2011). However, how best to develop physician leaders is unclear. There is a growing understanding that new, context-sensitive pluralistic leadership models are needed to meet the demands of increasingly complex organizations. Although there are many leadership theories and explorations in healthcare, collective leadership is gaining traction in the literature. Collective leadership refers to the collective actions of formal and informal leaders who act together to achieve organizational success (West *et al.*, 2014a, b; Contractor *et al.*, 2012; Fairhurst *et al.*, 2020). The literature on collective leadership comprises various theoretical strands including network leadership (Cullen-Lester and Yammarino, 2016), discursive leadership (Fairhurst, 2011), complexity leadership (Uhl-Bien *et al.*, 2007), and constructionist collective leadership (Ospina and Sorenson, 2006). These strands view leadership as an interactive, emergent process meant to develop group members' skill and adaptability in navigating complexity. An appealing concept, unfortunately a recent Cochrane review on collective leadership has revealed a need for more high-quality studies (Silva *et al.*, 2022).

In June 2020, the National Rehabilitation Hospital (NRH) transitioned to a new state-of-the-art building. The development of the new hospital presented a once in a lifetime opportunity to investigate and understand the lived experience of medical leadership in the organization and to explore ways to enhance leadership effectiveness to successfully navigate transition to a new hospital. There have been no published studies that have explored medical leadership experiences related to a hospital transition. The aim of this study was to explore the lived experiences of medical consultants, as part of the medical board, of leadership and leading prior to a move to a new hospital facility and to identify the perceived key elements necessary for effective collective medical leadership with the following research questions: 1. What is the lived experiences of medical consultants as part of the medical board? 2. What is the experience of medical leadership prior to a move to a new hospital facility? 3. What are the perceived key elements necessary for effective collective medical leadership?

## Methods

### Study design

This research is situated philosophically in a social constructionist ontology and epistemology where reality is socially constructed and is constantly in flux as it is continually renegotiated through experience (Berger and Luckmann, 2023) and where language is not viewed as a simple reflection of reality but implicit in the social production and reproduction of meaning and experience (Schwandt, 1998).

A qualitative research approach was chosen as the most appropriate method to gain a richer, deeper understanding of the phenomenon of medical leadership in complexity through the experiences of those who had directly experienced the phenomenon. This was guided by an examination of the extant literature on leading in complexity and examining the complexity of healthcare which recommends more qualitative approaches (Gardner *et al.*, 2020; Rosenhead *et al.*, 2019; Turgeon and Cote, 2000). Qualitative interviewing is a well-established flexible and powerful approach to capturing participants’ voices and through dialogue and interaction, meanings and understandings are created (Mason, 2017).

### Setting and context

The National Rehabilitation University Hospital (NRH) is the only complex specialist rehabilitation hospital in Ireland, a small island in North-West Europe with a population of 5 million. Ireland has a National Health Service provided by the Health Services Executive (HSE) and the NRH provides national comprehensive rehabilitation services to public patients following acquired brain injury, spinal cord injury or amputation. The hospital is overseen by a board of management and is funded by the HSE through a service level agreement which is agreed annually. With regard to clinical governance, the hospital adopted the clinical directorate structure negotiated in the Consultant Contract in 2008, with the first Clinical Director being appointed in 2015 (McAuliffe, 2014). The Clinical Director is appointed through a competitive process every 5 years. The hospital also has a Medical Rehabilitation programmatic structure, a requirement for accreditation through the Commission for Accreditation for Rehabilitation Facilities (CARF) with a Medical Director leading each of the five clinical programmes. In addition, the Hospital constitution requires the appointment of a Medical Board which is a subcommittee of the Hospital Board and is responsible to the Board of Management for clinical care, standards, and practice in the Hospital. The Clinical Director is a member of the medical board, and a chair is appointed by the hospital board. The role of chair is rotated through the membership of the medical board at 3 yearly intervals. Through the Chair of the Medical Board, who is a member of the Executive management team and board member, the Medical Board reports to and advises the Board of Management on all matters relating to clinical practice and any changes to that practice. The Medical Board is composed of all the members of the Consultant Medical Staff, including the Clinical Director and the Medical Directors and different medical board members are appointed to different hospital fora as required. Members also engage in leadership in informal roles and such matters are also shared at the medical board. Therefore, the medical board is the entity through which medical leadership is effected in the hospital and is a form of collective or shared leadership (Cullen-Lester and Yammarino, 2016; Yammarino *et al.*, 2012).

### Study population

As this was a study exploring medical leadership, all medical consultants who held substantive positions at the NRH and were members of the medical Board were invited to participate. These consultants are all specialists in Rehabilitation medicine (RM).

Those consultants who were not RM physicians or who had nominal sessional commitment (2 sessions or less) were excluded as they do not participate on the medical board or have leadership responsibilities in the hospital.

### Sampling

It was recognized that sample size is a contentious issue in qualitative research and the sample size was determined using the principle of data saturation with a minimum of six-twelve (Guest *et al.*, 2006; Braun and Clarke, 2013).

### Data collection

Following ethical approval from the hospital ethics committee and the approval of the Clinical Director (CD) and Chief Executive Officer (CEO), a group and individual e-mail invitation to participate in one-to-one interviews to explore the experience of medical leadership and the role of the medical board, was issued to the whole medical board (*n* = 20).

Prior to the scheduled interviews, the interview guide was shared as well as the outputs from two previous medical board away days (2016 and 2018), the hospital constitution and the terms of reference of the medical board as contained in the hospital constitution. Participants were informed that the key themes that would be generated from thematic analysis of the transcripts of the interviews would be shared at an away day to be arranged subsequently. Conscious of the importance of space and place in qualitative interviewing (Gagnon *et al.*, 2015), and the potential power imbalance by virtue of the researchers leadership role, the researcher allowed participants to choose the time, date and place for the interviews, and the researcher was flexible and available at the time and place that suited each participant. The researcher reflected on power relations at every stage of the process. The interviews were carried out over a four-week period.

Data was collected though semi-structured interviews (Alvesson and Deetz, 2000) carried out by the lead author who was Chair of the Medical Board at that time undertaking a DBA and exploring medical leadership in complexity. In keeping with Brinkmann and Kvale (Kvale and Brinkmann, 2009), the interviews were designed to obtain rich descriptions of the lived experience of interviewees in order to interpret the meaning of collective medical leadership in our organization. An interview guide was developed in accordance with the guidance proposed by Kvale and Brinkmann, containing the thematic research questions for the project and the interview questions (Supplementary file 1). These took into consideration both thematic and dynamic dimensions, to explore interviewees experience of medical leadership as enacted though the functions of the medical board and as individuals and to uncover any concerns regarding the move to the new hospital. Probes (open ended questions) were used to explore participants’ experiences of the medical board. Participants were encouraged to speak freely and were given the time and space to do so.

The interviews lasted 45–60 min, and were audio recorded and transcribed verbatim manually by the researcher with a non-identifying variable (P1, P2, etc.) assigned to each participant′s interview.

### Data analysis

According to Braun and Clarke (2006), thematic analysis is a method for developing analyzing and interpreting patterns across a qualitative dataset (Braun and Clarke, 2006). Thematic analysis is not tied to a particular theoretical outlook and so can be applied when using a range of theories and epistemological approaches Thematic analysis is an accessible, flexible, and increasingly popular method of qualitative data analysis. Thematic analysis involves the systematic process of coding to develop themes which is the ultimate analytic purpose of the research endeavor. Thematic analysis is a family of heterogeneous methods that have a common interest in patterns of meaning that are developed through a process of coding and theme generation. The data collected in this study was analyzed manually, and an inductive approach to analysis was utilized with data coding undertaken without a pre-determined coding frame which allowed the process to be driven by the actual data collected rather than any analytic preconceptions.

The six phases of thematic analysis described by Braun and Clarke were observed (Braun and Clarke, 2006):

1. Familiarization with data

The recordings were transcribed manually verbatim into word documents. The researcher immersed herself in the data by reading and rereading the transcripts many times until the researcher was familiar with the data, noticing and noting and critically reflecting on interesting patterns that might be relevant to the research questions.

2. Generating initial codes

Line by line coding of the interview transcripts was done manually with initial codes of interest related to the research questions highlighted in different colored highlighters. These evolved and expanded to develop a more comprehensive understanding of the underlying concept of medical leadership in the NRH.

3. Searching for themes

Common codes were initially grouped together due to frequency but then reorganized around patterns which created the initial, or candidate, themes that offered insights into the research questions. Braun and Clarke describe a theme as capturing *“something important about the data in relation to the research question and represents some level of patterned response or meaning within the data set*” (Braun and Clarke, 2006 p. 82). All the relevant coded data extracts within the identified candidate themes were collated.

4. Reviewing themes

These candidate themes were then reviewed in relation to the coded data and the entire data set and reorganized to ensure that they reflected something of importance about the data. Frequency of codes did not necessarily reflect an important pattern. During this phase, the researcher also reflected on their own values, beliefs, knowledge and biases.

5. Defining and naming themes

Through a further process of refinement, final themes and sub-themes were identified with each theme being unique and specific.

6. Producing the report

The process was written up and developed in PowerPoint presentation form and was shared with participants prior to and during a workshop where the findings were validated.

### Quality assurance

The study complies with the COREQ checklist for the explicit and comprehensive reporting of qualitative studies (Tong *et al.*, 2007). The interview guide was tested with the first interviewee to ensure the research questions were being answered and also to make sure the questions made sense to the participant. No amendments were required to be made following the test. Over the course of each interview, Schensul *et al.*’s three principles for ensuring the quality of the interviews were observed; Flow of the interviewee’s story was maintained, a positive relationship with the interviewee was maintained; and interviewer bias was avoided (Schensul *et al.*, 1999).

### Ethical clearance

The research was approved by the Hospital Research Ethics Committee and the Research ethics committee in UCD Ref: LS-E−20–09-Carroll. All participants were provided with a detailed information sheet and informed consent was obtained from them prior to the commencement of the interview. Participants had the right to withdraw at any time without explanation. De-identified transcripts were offered to be shared with participants for their approval prior to analysis and a post analysis report was shared with participants to ensure comfort with the quotes used. It was explained that although all efforts would be made to de-identify data, there was a remote possibility for them to be identified by other members of the organization. The researcher was aware of and attentive to the potential power differential between themselves and interview participants and addressed this by making the intent of the research clear to participants and allowing participants to choose the time and place of the interviews. Questions were shared before the interviews. Participants could view and edit interview transcripts before the researcher used them for analysis. Reflexivity was an important part of the data analysis, reflecting on the researcher’s values, beliefs, knowledge and biases. In addition the researcher gave participants an opportunity to read and comment on analyses before the researcher shared it with others through presentation.

## Results

All members of the medical board (*n* = 20) were invited by e-mail to participate in the semi-structured interviews. One e-mail address was incorrect, one participant was on sabbatical, and one was on maternity leave. Two participants sent written feedback, but this has not been included in this analysis as that would have required a different method of analysis. In total ten participants, all rehabilitation medicine consultants, participated in the semi structured interviews. Five were male and five female which was an unexpected finding as the specialty is predominantly female. There was also a reasonable distribution of new appointees (less than 10 years appointed *n* = 4) and more well-established participants (more than 10 years *n* = 6) which is in keeping with the distribution of the consultant body as a whole.

**Figure 1:**
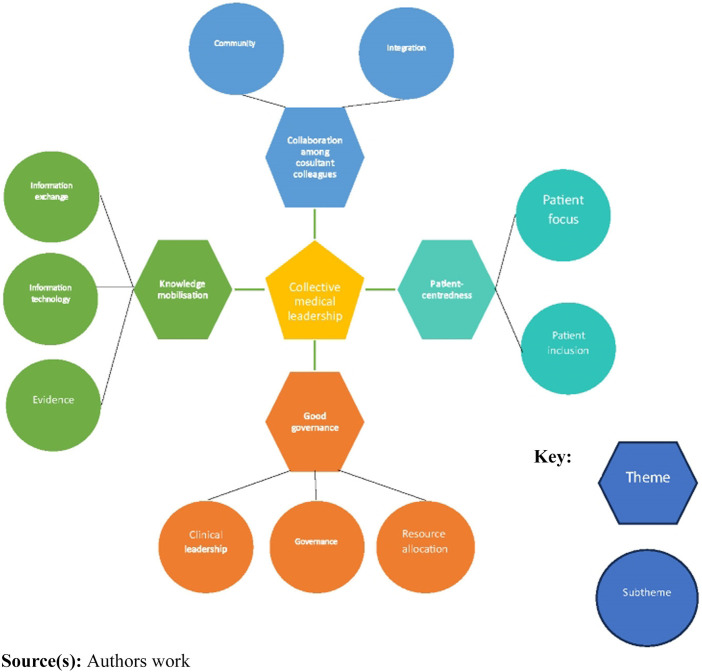
Thematic map

The inductive thematic analysis was performed in accordance with the six steps outlined by Brain and Clarke (2006) and Figure 1 shows the thematic map of the analysis.

Four overarching themes were generated though the process of inductive thematic analysis that illuminated the different dimensions of an overall conceptualization of collective medical leadership and addressed the research questions; medical consultants lived experience of collective leadership through the medial board (RQ1), the experience of medical leadership prior to the move to the new hospital (RQ2) and the perceived key elements necessary for effective collective medical leadership (RQ3), a reflection of the research questions. Where each theme addresses a specific research question is identified by RQ 1,2 etc.

### Theme 1: the importance of collaboration amongst consultant participants

This theme is comprised of the subthemes of community and integration and captures an overall narrative of a collective of senior medical colleagues leading teams and working together in an integrated manner with a shared purpose.

There was a sense that participants clearly valued the opportunity that medical board afforded them in coming together as a community and collective group (RQ1).

P10: “*It’s (medical board) good for participants to be able to come together to share understanding*” and P5: “*It’s (medical board) good for finding out about things that affect us all*”.

However, the data revealed that some participants felt that senior medical participants were not integrating or collaborating as well as they might (RQ2) with a participant (P2) expressing the view that *“I feel like an outlier*” and that communications especially e-mails were “*more like a bickering forum*”. Another stated (P4) “*I feel out of the loop of decision making”*.

Improving communication and promoting integration and relationships were identified as important by a number of participants (RQ3). P6: “*We need to maximise participation of all medical board members*” and P10: “*It would be good to have a forum, a supportive environment to discuss issues*”. A participant (P3) expressed the view “*there are such dominant voices and I wonder what contribution I can make*”. Participants also raised the challenge of time and conflicting demands and initiatives P2: *“We don’t have the time to make time for coming together”* and P5 “*Some of these so-called innovations end up causing a Dante’s inferno*”.

Some participants expressed feelings of isolation and exclusion and feelings of disillusionment (RQ1) as one participant (P10) stated: “*I am an outsider to the process. It is irrelevant to me*”. Another participant suggested self-management support as a mechanism to bring the medical body together (RQ3): (P9) “*Peer support is important perhaps we should have a Balint group otherwise how does self-care happen*?”.

### Theme 2: patient-centredness

Just as the previous theme encapsulates the need for collaboration as a core component of collective leadership, theme 2 focuses on the need for an organizing concept of patient centeredness. This theme is a synthesis of the subthemes patient focus and inclusion which participants felt was lacking in the current leadership structures and processes. There was a clear desire from participants for services to be more responsive to patients needs and more patient centered (RQ3) but there were concerns that nothing would change by the move to the new hospital and that the same issues would persist. One participant (P1) commented: “*nothing new – same issues, new building*”. Another (P7) stated: “*we might be in a shiny new building, but will patient care suffer?*”. One participant (P10) declared (in the context of not being able to get patients admitted): “*I am tired of apologizing to patients and staff in the acute hospitals for our unresponsiveness even though it’s not my fault. I feel like I’m fighting with everyone all the time*” (RQ2). Another participant (P4) asserted: “*there should be more focus on advocacy for our patients illustrating the challenges facing the patient and care givers*” and another (P6): “*The medical board needs to bridge the gap and ensure the patient voice is included in all major decisions*” (RQ3).

The central organizing concept for this theme is the participants’ articulation of the need to be more patient centered into the future.

### Theme 3: good governance

Governance was identified as a frequent code (*n* = 15). Through repeated engagement with the data, the researcher recognized that this was a complex and multifaceted code. The researcher interpreted differing conceptual understandings of governance and a conflation of clinical and corporate governance but there were clear expressions by participants of the need for clear lines of responsibility and accountability. The code was therefore promoted to a theme and was generated from the subthemes of clinical leadership, governance and resource allocation. The organizing concept for this theme is the latent sense of poor governance and the need for this to change. There was a general sense from the data that participants felt that the hospital needed to have a clearer governance structure with a clearly defined role for the medical board and a more effective executive management team (RQ3). It was felt that governance, and therefore leadership, was currently unclear (RQ1,2) with one participant (P7) describing it as “*fuzzy*” and “*mushy*” and with regard to the medical board (RQ1) *“what does it do anymore?”.* One participant (P2) wondered “*where are decisions made*? and “*who is leading who*?” (RQ1,2) and another (P5): “*There is a disconnect between the Executive and Medical Board, we have all the responsibility without the authority*” (RQ1) and P4: *“the* (hospital) *Executive don’t pick up leadership”* (RQ2*)*. Participants felt that governance could be clearer and that the move to the new hospital created opportunities for renewal (RQ3): P4 “ *It* (the move to the new hospital) *can be a dynamic time with new leadership*”. One participant (P7) stated “*The* (hospital) *constitution is at odds with the new clinical governance structures*” Another participant (P9) indicated “*we need to clarify the medical board’s purpose in the hospital*” (RQ1) and another (P6): “*we need to clarify the remit of the medical board*” (RQ2). Another stated (P4) “*there is lack of clarity of the role of the medical board and where it fits in vis-à-vis Clinical director – who has responsibility for what – blurred*” (RQ2) and another wondered (P1) “*Are we* (the medical board) *just a talking shop*?” (RQ1). One participant (P6) commented that it was always “*the same people putting themselves forwards for leadership roles*”. There was also a sense of futility and disillusionment of getting involved in change initiatives. As (P3) stated *“why bother designing if the designs aren’t implemented?”.*

However, participants really valued the leadership role of the medical board with P6 stating “*It bridges the gap between the clinical teams and the hospital board – it provides continuity*” (RQ1).

### Theme 4: knowledge mobilization

This theme was generated from many codes that related to data, ICT and information and evidence mobilization to support clinical leadership and innovation, a necessary component of the entrepreneurial leadership element of complexity leadership. An initial subtheme of information management was identified but as the researcher engaged with the data, knowledge mobilization emerged as a more reflective dynamic theme linking data and knowledge and people to enable better care for patients.

Creating opportunities for sharing different types of information was identified as important (RQ3) and that the absence of such opportunities as one participant put it (P7) “*it takes my goodwill away*” (RQ1). Another participant stated (P10): “*we need somewhere we can talk about stuff, you know, patients, a new journal paper and the football*” (RQ3). Another felt we needed to accelerate academic activities and teaching (P2): “*all the other hospitals have active academic departments – why don’t we*?” (RQ2). Another stated (P3): “*medical board has a major teaching and research role but no control of funding or separate budget – should we have a say in where the funding provided by the Universities for teaching by consultants goes?”* (RQ3). Participants felt that additional resources were required to support knowledge mobilization and improvement activities (P5) “*We cannot continue to do more with less – we’re starting to do less with less – we’re at a tipping point*” (RQ3).

## Discussion

This qualitative study revealed the lived experience of members of a medical board of effecting collective medical leadership in a National Rehabilitation Hospital prior to a move to a new hospital. Social constructionism is a sociological theory which relies on the development of understanding between people, leveraging different perspectives and experiences in the development of societal knowledge. In a constructionist view, knowledge is not obtained, nor created individually, instead it is shared, developed and contextualized based on cultural practices and group beliefs. Social constructionism views theory as generative, relational and practical (McNamee, 2014). Four key themes which influenced medical consultants’ experience of leadership were socially constructed through a process of inductive thematic analysis. These were: collaboration, patient centeredness, governance and knowledge mobilization. Various factors were identified that negatively influenced their leadership effectiveness.

These themes reflect the three essential aspects of collective leadership theory identified by Contractor and colleagues: people (the consultants, their teams, senior management and their relations), roles (the role of the medical board), and time (the developmental stages of teams) (Contractor *et al.*, 2012).

The themes also resonate with what Carson refers to as a positive internal team environment in his paper on shared, collective, leadership in teams (Carson *et al.*, 2007). According to Carson, internal team environment refers to the extent to which team members perceive the internal organizational climate to be supportive or unsupportive and is a function of three dimensions: shared purpose, social support, and voice.

Within the context of this research and the experience of the consultants, this equates with the medical team coming together in a supportive environment working together as a medical board with a shared common clear purpose and role and effecting shared leadership. This is also a core component of collective leadership theories which place great emphasis on the relational nature of leadership. The third component of Carson’s internal team environment is voice. In this research, this is reflected in participants desire as a medical board to participate actively in decision-making processes and advocate for patients and ensure that the patient’s voice is included in all major decisions.

Collaboration has been identified as a key component of collective leadership (Nightingale, 2020; West *et al.*, 2014a, b) but other authors have suggested that leadership disintegrates as a concept in collaborative settings (Denis *et al.*, 2012). This study supports the view that a move away from compartmentalization and siloed working toward more inclusive and collaborative approaches to problem solving and decision-making is required for the realization of change.

The literature suggests that leaders need to support their teams to shift from professional competition and towards a patient-centered and collaborative approach (Silva *et al.*, 2022) however little evidence exists about how best to develop such approaches (De Brún *et al.*, 2019). This study suggests that part of the solution may be in creating opportunities to meet and connect, which is aligned with what is suggested in complexity leadership theory by Uhl Bien and Arena (Arena and Uhl-Bien, 2016) for the creation of adaptive space in complex systems, a collective leadership theory that concentrates on the enablement of the learning, creative, and adaptive capacity of complex adaptive systems (CAS) (Uhl-Bien *et al.*, 2007).

The disconnect between clinical and corporate governance was also evident in the data and this disconnect has been often referred to in the literature (Adrian, 2000; Delaney, 2015; Flannigan, 2018) and participants were clear about the need to improve governance and decision making with clarity around roles and responsibilities. This is in keeping with an integrative governance approach which is defined by Deighan and Bullivant (2006) as “*systems, processes and behaviours by which healthcare organisations lead, direct and control their functions in order to achieve organisational objectives, safety and quality of service and in which they relate to patients and carers, the wider community and partner organisations*” (Deighan and Bullivant, 2006 p. 11). This also fits with the definition of clinical governance proposed by Som (2004) “*as a governance system for health-care organisations that promotes an integrated approach towards management of inputs, structures and process to improve the outcome of health-care service delivery where health staff work in an environment of greater accountability for clinical quality*” (Som, 2004 p. 89). However, these definitions do not give attention to the role of leadership which is a significant gap in healthcare literature yet the need for collaborative governance and collective leadership is well reflected in public leadership research (Ospina *et al.*, 2020).

Knowledge mobilization is a recognized component of the adaptive process, a component of complexity leadership theory (Arena and Uhl-Bien, 2016). Knowledge can be an element of each of the four components necessary for the creation of an adaptive space identified by Arena and Uhl Bien; the “4D” connections: discovery, development, diffusion, and disruption (Arena, 2018). Discovery connections connect people in a way that encourages exploration and curiosity. Development connections encourage the sharing and evolution of ideas. Diffusion connections facilitate the amplification of ideas and disruption connections remove barriers and enable innovation. Together these connections create a social construct that allows adaptation (Arena, 2021). Knowledge mobilization is also a key component of a learning organization defined by Watkins and Marsick as an organization that learns continuously and has the capacity to transform itself (Watkins and O’Neil, 2013). They propose seven dimensions of learning organizations: Continuous learning, inquiry and dialogue, team learning empowerment, embedded systems, system connection and strategic leadership.

This study embraced the recommendations of previous research to employ qualitative techniques to develop rich understandings of complex phenomena and achieved the aim of the study in that the research revealed insights into the lived experiences of leadership of medical consultants as part of a medical board, prior to a move to a new hospital facility and the research identified key elements necessary for effective collective medical leadership.

## Conclusion

Modern leadership theory has shifted from the traditional hierarchical and authoritative leadership model to one that is inherently relational and collective. Leadership is recognized as an important factor in shaping organizational culture and achieving better experiences of care for patients and improved experience of staff. Therefore, ensuring the requisite structures and processes exist to support leadership development and practice is vital.

### Implications for policy and practice

This research has provided valuable insights into the experience of collective leadership of a medical board and has identified important factors to enable optimal medical leadership in complex systems that could support healthcare professionals, policy makers and researchers’ ability to plan effective collective leadership interventions. These include collaboration, clear integrated governance, person centeredness and the importance of knowledge mobilization. By developing the necessary structures and processes indicated in this study, organizations could confidently move into a new facility and into a new future delivering the high quality, person centered care that is their collective mission.

### Implications for methodology and theory

This study makes a number of contributions to methodology and collective leadership theory. This is the first use of inductive thematic analysis to explore collective medical leadership and shows how qualitative approaches can be powerful approaches to understanding complex phenomena. The literature has shown that although collective leadership theory is gaining traction in healthcare little consensus exists about how to conceptualize, define or measure collective leadership. This study identifies four key areas that influence collective leadership that can be used to explore and design interventions and strengthen the evidence base for collective leadership theory.

### Limitations and future research

Only ten consultants participated so it could be argued that saturation was not achieved. However, there is ambiguity in the literature about how many interview are required and the researcher was able to interpret very similar themes across the datasets. Participants validated the findings at a workshop (member validation) (Seale, 1999).

How generalizable these findings are is questionable because of the unique context in which the research took place. However, in keeping with the cases made by Lewis *et al*. (2003) and Chenail (2010), the findings do reveal useful information about the phenomenon of collective medical leadership and although the contextual orientation of this research, a hospital move, is a very rare event, it is likely that the findings are transferable to other medical collectives and situations (Lewis *et al.*, 2003; Chenail, 2010).

Further empirical research is required on how collective medical complexity leadership can be supported and developed.

## Supplementary file

### Interview guide

The design of the research interview followed the seven stages of interview inquiry described by Kvale (1998).

### Thematic research questions

1. What is the lived experiences of medical consultants as part of the medical board?
2. What is the leadership style and leading of medical consultants prior to a move to a new hospital facility?
3. What are the perceived key elements necessary for effective collective medical leadership?

### Semi-structured interview guide

The researcher will have a reflective and adaptative approach during the conversation and have some be flexibility to react to new information and discoveries during the interview to get in-depth understanding or for clarification.

1. Can you tell me how do you feel the Medical Board is functioning currently?
2. What do you think is working well?
3. What do you think is not working well?
4. What suggestions do you have to make things better?
5. How do you feel about the move to the new hospital?
6. How might any concerns be addressed?
7. Have you any other thoughts you’d like to share?

Possible follow up question prompts:

What happened in the episode mentioned?

Could you say something more about that?

Can you give a more detailed description of what happened?

Do you have further examples of this?

If a theme is exhausted by breaking off long irrelevant answers: “I would now like to introduce another topic: …”

Allow Silence: By allowing pauses the interviewees have ample time to associate and reflect and break the silence themselves.

Interpreting questions: “You then mean that ….?” “Is it correct that you feel that … ?”Does the expression …. Cover what you have just expressed?”
